# Comparative Evaluation of Micro Tensile Bond Strength and Microleakage of Ionoseal Glass-Composite as a Fissure Sealant Material, Following Four Different Enamel Surface Pretreatments

**DOI:** 10.30476/DENTJODS.2021.91093.1558

**Published:** 2022-12

**Authors:** Samaneh Hesami, Davood Ghasemi, Shahriar Shahriari

**Affiliations:** 1 Postgraduate Student, Dept. of Pediatric Dentistry, School of Dentistry, Isfahan (Khorasgan) Branch, Islamic Azad University, Isfahan, Iran; 2 Dept. of Pediatric Dentistry, School of Dentistry, Isfahan (Khorasgan) Branch, Islamic Azad University, Isfahan, Iran; 3 Dept. of Operative Dentistry, School of Dentistry, Isfahan (Khorasgan) Branch, Islamic Azad University, Isfahan, Iran

**Keywords:** Fissure sealant, Composite, Microleakage

## Abstract

**Statement of the Problem:**

Sealants are employed to prevent carious lesion initiation and to arrest caries progression by providing a physical barrier that inhibits accumulation of microorganisms and food particles in pits and fissures. The two most common materials used for sealing pits and fissures are resins and glass-ionomers. Ionoseal from VOCO company is one of the light curing glass-ionomer composite cements, whose mechanical properties should be investigated.

**Purpose:**

The aim of this study was to compare the micro tensile bond strength and micro leakage of Ionoseal with different surface pretreatments.

**Materials and Method:**

This in vitro experimental study was conducted on five groups of 95 sound human premolars. Each group consisted of five teeth for the micro tensile test and 14 teeth for the micro leakage test. The groups regarding the materials and the methods used were defined as Group 1: 35% phosphoric acid + total etch adhesive + Ionoseal, Group 2: universal adhesive+Ionoseal, Group 3: 35% phosphoric acid + Ionoseal,Group 4: Ionoseal, and Group 5 (control group): 35% phosphoric acid+Embrace fissure sealant. On the pre-pared buccal enamel of each tooth, a 5 mm block of the sealing material was created and thermo cycled. The specimens were serial sectioned to a cross section of about 1×1mm and loaded in a tensile pressure (0.5 mm/min) until failure occurred. For the micro leakage test, the sealing material was placed into the prepared occlusal fissures and teeth were thermo cycled, and then immersed in 5%methylene blue. A section was made buccolingually; the dye penetration rate was measured based on Williams and Winter criteria under a stereomi-croscope. Data was analyzed with one-way ANOVA test and Kruskal-Wallis test.

**Results:**

Mean micro tensile bond strength was significantly different between the groups (*p*< 0.001), and was significantly higher in Group 1. There was no significant difference between the frequency of modes of failure (*p*= 0.81). The rate of micro leakage was significantly different between the five groups (*p*< 0.001) and in Group 1 and 3; it was significantly lower than the other three groups.

**Conclusion:**

Ionoseal can be used successfully as a fissure sealant material. Etching the enamel surface with phosphoric acid is necessary and the use of a bonding agent before Ionoseal placement improves results.

## Introduction

In recent decades, significant advances in caries prevention have been made due to a number of factors, including fluoride intake, improved responsiveness to the benefits of early care, increased admissions to dental care, more financial coverage by insurance companies, and government-sponsored programs to prevent and repair restorative teeth for children [ 1 ]. Regardless of these efforts, dental caries is yet considered as the most common chronic childhood disease [ 1 ]. Approximately 56% of children aged 6 to 8 years old have caries in the deciduous teeth and 21% of children aged 6 to 11 years have experienced caries in permanent teeth [ 1 ]. 

Sealants are used to prevent the onset or to stop the progression of caries with the aim of providing a physical barrier that prevents the accumulation of food particles and microorganisms in the pits and fissures [ 2 ]. Children and adolescents receiving sealants on healthy occlusal surfaces or carious lesions without pit cavities and fissures in deciduous and permanent molars showed a 76% reduction in the risk of new caries within two years of follow-up compared to control groups [ 1 ]. Even after seven years or more of follow-up, children and adolescents with sealants showed a 29% incidence of caries compared to 74% incidence of caries in the non-sealant group [ 1 ].

The most common materials used to seal pits and fissures are resins and glass ionomers. It has been shown that both of these materials have the potential to prevent caries [ 3 ]. In 1970, Bonocore introduced the resin Bisphenol-a-glycidyl methacrylate, known as BIS-GMA [ 4 ]. It was used as a base for many sealants and composites due to its resistance to bacterial decomposition and the formation of a durable bond with etched enamel. In 1974, glass ionomer cements (GICs) were introduced by Mclean and Wilson to seal dental grooves [ 4 ]. Resin-modified glass ionomers (RMGICs) were introduced with the attempt of overcoming problems such as moisture sensitivity and weak physical properties of conventional glass ionomers. RMGIC enhanced the physical properties of conventional glass ionomers while preserving clinical benefits like adhesion and fluoride release and providing some protection against caries [ 5 ].

VOCO has introduced Ionoseal (Ionoseal, VOCO GmbH, Cuxhaven, Germany) as a light-curing glass ionomer composite [ 6 ]. Ionoseal has been shown to be highly moisturizing, making it more convenient to use in hard-to-reach areas. This material is highly favorable for the dentist to use and, as claimed by the manufacturer, has great mechanical properties, high compressive strength, and biocompatibility of the product is sustained by the concurrent release of fluoride. Moreover, this material can be cured by light in a few seconds; therefore, its application is effectively time- saving [ 6 ]. It has been shown that the microleakage of this material is equal to or even less than the conventional materials used for fissure sealants. It has also been indicated that regarding the high amount of micro hardness of Ionoseal, its ease of use, and timesaving benefits, it is a good option as a reliable restorative material for dental care in children [ 6 ]. Ionoseal can be applied on enamel without surface preparation with phosphoric acid as recommended by the manufacturer. Ease of use of this material and elimination of etching and rinsing steps in the pediatric dentistry is very important. Especially in non-cooperative children, and due to the importance of isolation in the success of fissure sealant, rapid clinical application of a sealant is very important. Thus, this material could provide reduced working time and greater success as well as greater parent satisfaction [ 6 ].

McMurphy *et al.* [ 2 ] showed that adding bonding material without curing before sealant therapy reduces the micro tensile bond strength and the exposure to the thermo cycling had no effect on the micro tensile bond strength. In a study by Nahvi *et al.* [ 7 ], it was shown that the use of bonding significantly reduced the micro leakage of fissure sealants.

Due to the lack of sufficient studies on the application of Ionoseal as a fissure sealant and because of its excellent advantages, such as ease of use, faster work process, and suitable physical and mechanical properties, this study was designed to evaluate the micro leakage and micro tensile bond strength of Ionoseal in four different surface preparations.

## Materials and Method

The present laboratory experimental study was registered with the Ethics index IR.IAU.KHUISF.REC.1398. 122. In this study, 95 human premolars, extracted for orthodontic purposes, which had no structural defects, cracks, or fractures and were collected. For disinfection, the teeth were brushed and then immersed in chloramine 0.5% T solution for one week [ 8 ]. After keeping the teeth in distilled water at room temperature, the study groups were determined in the following order: 

The groups regarding the materials and the methods used were defined as First: 35% phosphoric acid+Total H adhesive+Ionoseal, Second: Universal adhesive+ Ionoseal, Third: 35% phosphoric acid+ Ionoseal, Fourth: Ionoseal, Fifth: 35% phosphoric acid+ Embrace fissure sealant. After determining the study groups, the samples were prepared and examined in two parts: micro tensile bond strength and micro leakage. The materials used are shown in Table 1.

**Table 1:** The materials used for this study

Ionoseal	VOCO, GmbH, Cuxhaven, Germany
Solobond M	VOCO, GmbH, Cuxhaven, Germany
Futurabond U	VOCO, GmbH, Cuxhaven, Germany
Embrace WetBond	Pulpdent, Watertown, MA
Etching gel (Vococid)	VOCO, GmbH, Cuxhaven, Germany

### Preparation of samples for micro tensile bond strength

Twenty-five teeth were selected and their roots were removed from the CEJ area by a non-stop cutting machine and then randomly divided into five groups. Each of the four experimental groups consisted of five teeth with surface preparation before applying Ionoseal according to the manufacturer's instructions, and the control group consisted of five teeth (n=5) in which surface preparation was done and conventional fissure sealant was applied.

In Group 1, the enamel of the buccal surface of the five teeth was etched with 35% phosphoric acid for 20 seconds, then rinsed with a water spray for 10 seconds, and dried with air spray for five seconds. Solobond M adhesive, a fifth-generation bonding system, was then applied to the etched enamel. According to the manufacturer's instructions, after 30 seconds of adhesive application, it was sprayed with air and then cured for 20 seconds by a LED light curing device. 

In Group 2, on the enamel of the buccal surface of the five samples, the Universal Adhesive (Futurabond U) was applied according to the manufacturer's instructions without the use of acid etching. The adhesive was applied to the enamel by a micro brush for 20 seconds, then sprayed with air for five seconds, and cured for 10 seconds. 

In Group 3, the enamel of the buccal surfaces of the five teeth were etched with 35% phosphoric acid for 20 seconds, then rinsed with water spray for 10 seconds, and dried with air spray for five seconds.

In Group 4, no preparation was performed on the buccal enamel surface of the samples. Ionoseal was applied on the enamel of the buccal surface of the prepared teeth in the study (first to fourth) groups by using a plastic mold with a height and diameter of 5 mm. Then it was cured in layers with thickness of less than 2 mm for 20 seconds. 

In Group 5 (control group), the enamel of the buccal surfaces of samples were etched with 35% phosphoric acid for 20 seconds, then rinsed with water spray for 10 seconds, and dried with air spray for 5 seconds. Then a 5mm block of fissure sealant material (Embrace Wet Bond sealant) was placed on the etched enamel by a plastic mold. The fissure sealant was placed on the enamel each time in a thickness of less than 2mm and each layer was cured for 20 seconds.

The samples were subjected to 500 thermal cycles between 5 and 55°C. Each cycle consisted of 20 seconds in hot water (55°C), 20 seconds in cold water (5°C), and 10 seconds to transfer from one source to another.

Each sample prepared in a plastic mold was mounted by a transparent resin and then transferred to a fully automatic cutting machine. The samples were cut to obtain slices with a cross-sectional area of ​​1mm2. A digital caliper was used to ensure the dimensions of the specimens. A large number of specimens were obtained from each tooth; however, only 20 healthy specimens were prepared in each group. Due to the adhesive failure of all Group 4 samples, this group did not enter the micro tensile bond strength test. Samples of each group were kept separately in an incubator at 37°C for 24 hours before being connected to the micro tensile bond strength-measuring device.

To measure micro tensile bond strength, the prepared samples prepared were attached to the plates of micro tensile machine (MTD-500 plus- SD Mechatronik, Germany) by cyanoacrylate adhesive. The specimens were then subjected to a tensile force at 0.5mm/min until they broke. The force at which the failure occurred was recorded in Newtons. The amount of micro tensile bond strength was obtained by dividing the force in Newton's by the cross-sectional area of ​​the specimen [ 2 ]. The type of fractures in each sample was determined under a stereomicroscope with a 40× magnification and the fractures were divided into adhesive and cohesive in material and cohesive in teeth [ 8 ].

### Preparation of samples for micro leakage testing

Seventy teeth were randomly divided into five groups with 14 premolars in each group. The pits and fissures of the occlusal surfaces of these samples were prepared similar to the previous groups. The samples were then subjected to thermo cycling using the micro tensile bond strength method mentioned above. After thermo cycling, the samples were prepared to be placed in a color solution. The root ends of the teeth were blocked with wax and covered with two layers of nail polish up to two millimeters from the sealant edge to minimize the penetration of paint from other parts of the tooth. The teeth were then incubated in 5% methylene blue for 24 hours at 37°C to allow the paint to penetrate the space between the enamel and the fissure sealant. The samples were then thoroughly rinsed to remove excess paint. 

Buccolingual parallel cuts were made in the direction of the longitudinal axis of the tooth and in the center of the mesiodistal width by a device with a diamond blade cooled by water. Thus, in each group, 28 samples were obtained to evaluate micro leakage. The samples were examined under a stereomicroscope with 40× magnification and then they were photographed under a microscope. The resulting color penetration was divided according to the Williams and winter index shown in Table 2. An observer categorized the color penetration while blinded to the types of sample [ 8 ].

**Table 2:** Williams and Winter index for color penetration [7]

Grades	Dye penetration
Grade 0	No dye penetration between the tooth surface and the sealant
Grade 1	Dye penetration into less than one third of the entire length of the surface between the sealant and the tooth structure
Grade 2	Dye penetration into one third to two thirds of the entire length of the surface between the sealant and the tooth structure
Grade 3	Dye penetration into more than two thirds of the entire length of the surface between the sealant and the tooth structure

Data were analyzed using one-way ANOVA test and Kruskal-Wallis test and SPSS25 software.

## Results

Due to the adhesive failure of all samples in the fourth group, this group did not enter the micro tensile bond strength test. Kolmogorov-Smirnov test showed that the bond strength in all groups followed the normal distribution. Therefore, one-way analysis of variance was used to compare bond strength between the four groups.

The one-way analysis of variance test showed that the mean bond strength was significantly different between the four groups (*p*< 0.001). The Tukey post hoc test showed that the average bond strength in the first group was significantly higher than the second two groups (*p*< 0.001), third (*p*= 0.02) and fifth (*p*< 0.001). The mean bond strength in the third group was significantly higher than the second group (*p*= 0.004), but there was no significant difference between the fifth group and the second two groups (*p*= 0.10) and the third (*p*= 0.19). The average bond strength is shown in Table 3. The chi-square test with a likelihood ratio showed that the frequency distribution of the fracture type was not significantly different between the four groups (*p*= 0.81) (Table 4). The Kruskal-Wallis test showed that the amount of microleakage was significantly different between the five groups (*p*< 0.001) (Table 5). The Mann-Whitney test showed that the amount of microleakage in groups 1 and 3 was significantly lower than the other three groups. There was no significant difference between the two groups 1 and 3 and between the three groups 2, 4 and 5 (Table 6). 

**Table 3:** Average bond strength in different groups

Groups	Average	Standard deviation	*p* Value
First	17.3	4.2	<0.001
Second	10.1	2.9	
Third	14.1	5.3	
Fifth	12.4	4.02	

**Table 4:** Frequency distribution of fracture type in different groups

Groups	Adhesive		Mix		Cohesive		*p* Value
	N	Percentage	N	Percentage	N	Percentage	
First	8	40	4	20	8	40	0.81
Second	13	65	2	10	5	25	
Third	11	55	2	10	7	35	
Fifth	10	50	3	15	7	35	

**Table 5:** Frequency distribution of microleakage in different groups

Groups	0		1		2		3		*p* Value
	N	Percentage	N	Percentage	N	Percentage	N	Percentage	
First	21	75	5	17.9	2	7.1	0	0	& 0.001
Second	2	7.1	13	46.5	6	21.4	7	25	
Third	21	75	4	14.3	3	10.7	0	0	
Fourth	6	21.4	11	39.3	4	14.3	7	25	
Fifth	5	17.9	4	14.3	10	35.7	9	32.1

**Table 6:** Comparison of the frequency distribution of microleakage between two groups

Groups	*p* Value
First & Second	<0.001
First & Third	0.94
First & Fourth	<0.001
First & Fifth	<0.001
Second & Third	<0.001
Second & Fourth	0.40
Second & Fifth	0.42
Third & Fourth	<0.001
Third & Fifth	<0.001
Fourth &amp; Fifth	0.18

## Discussion

The long-term clinical success of fissure sealants is strongly interrelated to their proper employment. A dry surface of enamel is essential to achieve proper adhesion [ 9 ]. Therefore, the use of materials with less sensitivity to moisture and easier application techniques is very important, especially in difficult isolated conditions [ 9 ].

VOCO has introduced Ionoseal as a glass ionomer composite cement with high compressive strength and biocompatibility that can be used quickly (cured by light in a few seconds) as a suitable material for fissure sealants [ 6 ]. Moreover, Ionoseal can be applied on tooth enamel without surface preparation as recommended by the manufacturer. According to the advantages of Ionoseal, in this study, the physical properties of this material have been investigated using two different application methods (micro tensile bond strength and microleakage).

The clinical success of dental sealants is associated with their ability to adhere firmly to the enamel surface and to separate pits and fissures from the oral environment safely [ 10 ]. In the present study, the micro tensile bond strength was measured for the first, second, third, and fifth groups. In the fourth group (using Ionoseal without surface preparation on enamel); all samples failed as adhesive type during the preparation process. It seems that the strength of Ionoseal bond on enamel without surface preparation is not enough to withstand the stress of the sample preparation process (slices with a cross section of 1×1 mm). The highest bond strength was seen in the first group (total etch adhesive and Ionoseal) and was significantly higher than the other groups. The results are in line with the findings of many studies, including that of Papacchini *et al.* [ 10 ], Pouyanfar *et al.* [ 8 ], and Autio-Gold *et al.* [ 12 ], in which the use of a total etched adhesive between fissure sealant and etched enamel was suggested to increase adhesion and compatibility. However, in some studies such as the study of Murphy *et al.* [ 2 ] as well as the study of Bagheri *et al.* [ 13 ], the use of adhesives before sealant application did not significantly improve bond strength.

Also, the average bond strength of the third group (etch and Ionoseal) was significantly higher than the second group (self-etch and Ionoseal). In fact, the lowest bond strength among the four mentioned groups (first, second, third, and fifth groups) belonged to the second group, and this result confirms the results of previous studies such as the study of Pouyanfar *et al.* [ 8 ], in which the use of universal adhesives and other self-etch adhesives were not recommended on enamel without prior etching. However, in their study, there was no significant difference between the micro tensile bond strength of the universal adhesive without etching comparing to other two-stage and three-stage, etch and rinse, and self-etching adhesives, although when enamel was etched, the universal adhesive had the highest bond strength among the other groups [ 8 ].

In the study of Papacchini *et al.* [ 10 ], the micro tensile bond strength of the groups containing Fuji VII glass ionomer and Fuji II light cure RMGI was lower than the groups containing resin fissure sealant, which is different from the present study. This difference might be due to Ionoseal compounds, which are a composite glass ionomer, in comparison with other RMGIs [ 10 ]. In addition, in the present study, 35% phosphoric acid was used for surface preparation, while the mentioned study used 20% polyacrylic acid solution. It should be noted that 35% phosphoric acid is stronger and probably has better etching on the intact surface of the enamel. Therefore, the Ionoseal resin components showed better retention and bond strength to enamel by creating deeper tags.

In the study of Panigrahi *et al.* [ 14 ], the bond strength of Embrace fissure sealant to enamel was reported to be 21.720 MP, which is much higher than the value obtained in the present study. The reason for this difference might be the use of different teeth (molars) and fewer specimens (n=10) compared to the present study (n=20).

In this study, sealant therapy was performed without enameloplasty, because according to previous studies [ 15 ], the difference in fissure depth does not cause a significant change in microleakage. The results of the present study showed that the amount of microleakage in the first group (total-etch adhesive and Ionoseal) and the third group (etch and Ionoseal) was significantly lower than the other groups.

In the study of Khodadadi *et al.* [ 16 ], the use of Ionoseal without etching and bonding showed higher microleakage than surface preparation with acid etch and the application of total-etch adhesive. However, application of Ionoseal with acid etch and bonding agent presented no significance difference with other samples in which fissure sealants and flowable composites were applied with etching and bonding.

In the present study, the microleakage of the third group (etch and Ionoseal) was significantly lower than the control group due to the dual bonding mechanism of Ionoseal with the tooth structure (acid-base reaction of glass ionomeric component + polymerization of resin component). This can reduce the stress between the material and the tooth and show better adhesion and adaptation.

When the Ionoseal was placed on the enamel without any surface preparation (Group 4) and bonded only through the glass ionomer component, the resulting microleakage was no different from Embrace fissure sealant.

The reduction of Ionoseal microleakage after the use of acid etching in the present study confirms the results of the Lodlow *et al.*’s study [ 17 ] that reported the reduction of microleakage when RMGI was used after selective etching of the enamel. The results of the study of Nahvi *et al.* [ 7 ] showed that the use of bonding agent along with the usual method of fissure sealant significantly reduced microleakage. In addition, the use of self-etch bonding agent was reported to be less effective in reducing microleakage compared to using the acid etching method with the bonding agent [ 7 ]. In the present study, the use of universal self-etch bond with Ionoseal showed the weakest results in bond strength and microleakage, which is in line with the results of the study of Hannig *et al.* [ 15 ]. However, in the study of Nahvi *et al.* [ 7 ], regardless of the type of adhesive system, the amount of microleakage was reduced by adding a bonding agent, which is different from the present study. In the study of Morales *et al.* [ 18 ], the use of bonding agent with acid etch significantly reduced the microleakage of fissure sealants compared to the usual method or the self-etch adhesive (in accordance with the present study), but the self-etch adhesive and the conventional method of fissure sealant were not significantly different in terms of microleakage. However, in the present study, the use of self-etch adhesive increased the microleakage compared to the conventional method of fissure sealant with Ionoseal. Nevertheless, there was no significant difference in microleakage compared to the usual method of fissure sealant and Embrace fissure sealant. It should be noted that in the mentioned study, enameloplasty was performed, which could be effective in obtaining better sealing by way of self-etch adhesive.

Future clinical studies are suggested to evaluate the success of Ionoseal as a fissure sealant in natural oral conditions with larger samples and variables.

## Conclusion

Ionoseal can be used as a successful fissure sealant in terms of bond strength and microleakage. Etching of the enamel surface with phosphoric acid is necessary for the use of Ionoseal as a fissure sealant. Moreover, applying an adhesive after etching and before the use of Ionoseal is associated with better results.

## Conflict of Interest

The authors declare that they have no conflict of interest.
